# Ultrasound-Guided Block of the Internal Branch of the Superior Laryngeal Nerve Reduces Postoperative Sore Throat Caused by Suspension Laryngoscopic Surgery: A Prospective Randomized Trial

**DOI:** 10.3389/fsurg.2022.829811

**Published:** 2022-02-15

**Authors:** Yin Bao, Jun Xiong, Huijun Wang, Yang Zhang, Qi Zhong, Guyan Wang

**Affiliations:** ^1^Department of Anesthesiology, Beijing Tongren Hospital, Capital Medical University, Beijing, China; ^2^Department of Anesthesiology, Shenzhen University General Hospital, Shenzhen University, Shenzhen, China; ^3^Key Laboratory of Otolaryngology Head and Neck Surgery (Ministry of Education of China), Department of Otolaryngology Head and Neck Surgery, Beijing Tongren Hospital, Capital Medical University, Beijing, China

**Keywords:** ultrasound, superior laryngeal nerve, suspension laryngoscope, postoperative sore throat, cough

## Abstract

**Objective:**

Suspension laryngoscopy is commonly used in operative laryngology. Although it is efficient and minimally invasive in most cases, it can lead to postoperative sore throat (POST) and cough. Because of intensive stimulation by the rigid metal suspension laryngoscope, procedures must be implemented under general anesthesia. Together, these factors increase the possibility of postoperative complications. Blocking the internal branch of the superior laryngeal nerve (SLN) is useful in inhibiting the endotracheal intubation stress response. Thus, we evaluated the efficacy of ultrasound-guided block of the internal branches of the superior laryngeal nerve to improve postoperative complications.

**Methods:**

A total of 64 patients was scheduled for elective laryngeal cancer resection, and suspension laryngoscopic surgery was performed under general anesthesia with a block of the internal branch of the superior laryngeal nerve (group iSLNB, *n* = 32) and without a block (group C, *n* = 32). Patients in group iSLNB received ultrasound-guided blocks of the internal branches of superior laryngeal nerve bilaterally (0.2% ropivacaine, 2 ml each side). The primary outcome measures were the incidence and severity of sore throat and cough assessed 0.5, 2, 6, and 24 h after tracheal extubation. The secondary outcome measures were heart rate and mean arterial pressure on arrival in the operating room (T0), at endotracheal intubation (T1), upon insertion of the suspension laryngoscope (T2), 5 min after insertion (T3), at tracheal extubation (T4), and 5 min after extubation (T5). Blood glucose levels were measured at T0, T3, and T5.

**Results:**

The incidence and severity of POST and cough in the iSLNB (internal branch of superior laryngeal nerve block) group were significantly reduced within 6 h after extubation compared with those in the control group, regardless of whether swallowing was present (*P* < 0.05). Compared to the control group from T0–T5, except at T0, the iSLNB group had significantly lower heart rate and mean arterial pressure (*P* < 0.05). Compared to T0, at other time points, the heart rates in the control group were significantly increased (*P* < 0.05), and the mean arterial pressures at other time points in the iSLNB group were significantly lower than those at T0 (*P* < 0.05). The blood glucose levels at T3 and T5 in the iSLNB group were significantly reduced compared with those in the control group (*P* < 0.05).

**Conclusion:**

Ultrasound-guided block of the internal branch of the superior laryngeal nerve might effectively ameliorate postoperative complications secondary to suspension laryngoscopic surgery with endotracheal intubation under general anesthesia and improve hemodynamic stability.

**Clinical Trial Registration:**

https://www.chictr.org.cn, identifier: ChiCTR2100049801.

## Introduction

Transoral surgery is now an important strategy in the treatment of laryngeal–pharyngeal cancer because it is less invasive than traditional open procedures (1), in which laryngeal exposure is one of the most limiting factors (2). A specially designed suspension laryngoscope has to be inserted to create a working space for head–neck surgeons in the pharyngeal lumen. This procedure is typically implemented under general anesthesia, as the rigid metal laryngoscope can produce significantly strong stimulation (3). This stress causes an increase in blood pressure and heart rate that is sustained non-transiently in laryngeal–pharyngeal cancer transoral operations; it may result in a detrimental hemodynamic response in patients, particularly cardiac and neurosurgical patients (4). It also produces a sustained postoperative sore throat (POST) in the majority of patients, which might persist for 3 postoperative days or longer and increase the use of prescription analgesics and narcotics (5). Although medicines, devices, and techniques are being developed to ameliorate the incidence of POST, these interventions only result in a minor reduction in its severity (6).

The superior laryngeal nerve (SLN) is not a pure motor nerve (7) and has different functions, one of them being the supraglottic sensitivity. The internal branch provides sensory innervation to the larynx above the vocal folds, which enters the thyrohyoid membrane with the superior laryngeal artery (8). Blocking the SLN could inhibit the reflexive response caused by foreign body stimulation of the larynx and glottis; thus, superior laryngeal nerve block (SLNB) is frequently applied in awake endotracheal intubation of potentially difficult airways to alleviate patient discomfort and to prevent airway obstruction due to stimulation of the pharynx (9, 10). Meanwhile, SLNB or block or transection of its internal branch is a viable therapeutic option for medically refractory neuropathic cough (8, 11). However, SLNB, by using a local anesthetic as an injection, might cause complications, such as intravascular injection, nerve damage (12), and dysphagia, and even more serious complications including blindness or upper cranial nerve neuropathies (13).

Currently, ultrasound is widely used in a multitude of clinical applications. With precise location of the target nerve and anatomic marks, ultrasound-guided nerve block brings potential advantages, including a higher success rate and shorter performance time; more importantly, ultrasound visualization can reduce relevant complications (14). Therefore, we applied an internal branch of the SLN block (iSLNB) with ultrasound visualization.

Superior laryngeal nerve block is now commonly used in endotracheal intubation and bronchoscopic maneuvers, which result in relatively transient stimulations, so SLNB performed by lidocaine injection is the first choice in these clinical applications (13, 15). These are obviously different from patients with laryngeal cancer undergoing suspension laryngoscopic surgery, whose operative process has a longer duration and causes stronger stimulation. Therefore, we implemented iSLNB with ropivacaine to evaluate its intra and postoperative effects. We hypothesized that ultrasound-guided iSLNB with ropivacaine combined with general anesthesia would ameliorate the severity and incidence of POST and cough, as well as stabilize the hemodynamic response in patients undergoing suspension laryngoscopic surgery.

## Materials and Methods

### Study

This prospective randomized blind clinical trial was conducted after receiving the ethics committee approval (TRECKY-2021-139), and all enrolled patients signed written informed consent forms. The present study was registered prior to patient recruitment at the Chinese Clinical Trial Registry (https://www.chictr.org.cn, Yin Bao) on August 9, 2021, and the registration number was ChiCTR2100049801.

### Patients

A total of 64 patients scheduled for laryngeal cancer resection by suspension laryngoscopic surgery under general anesthesia were recruited at this tertiary hospital from September to October 2021. The inclusion criteria were as follows: American Society of Anesthesiologists (I–II) patients with laryngeal cancer (T1-2N0M0) aged 35–65 years and the ability to cooperate with postoperative follow-up. All recruited patients received laryngeal cancer resection with a CO_2_ laser. The exclusion criteria were as follows: coagulation disorder, patients on analgesics, hypersensitivity to the study medicine, patients requiring awake intubation due to a difficult airway, and severe cardiovascular and respiratory dysfunction.

**Figure 1:**
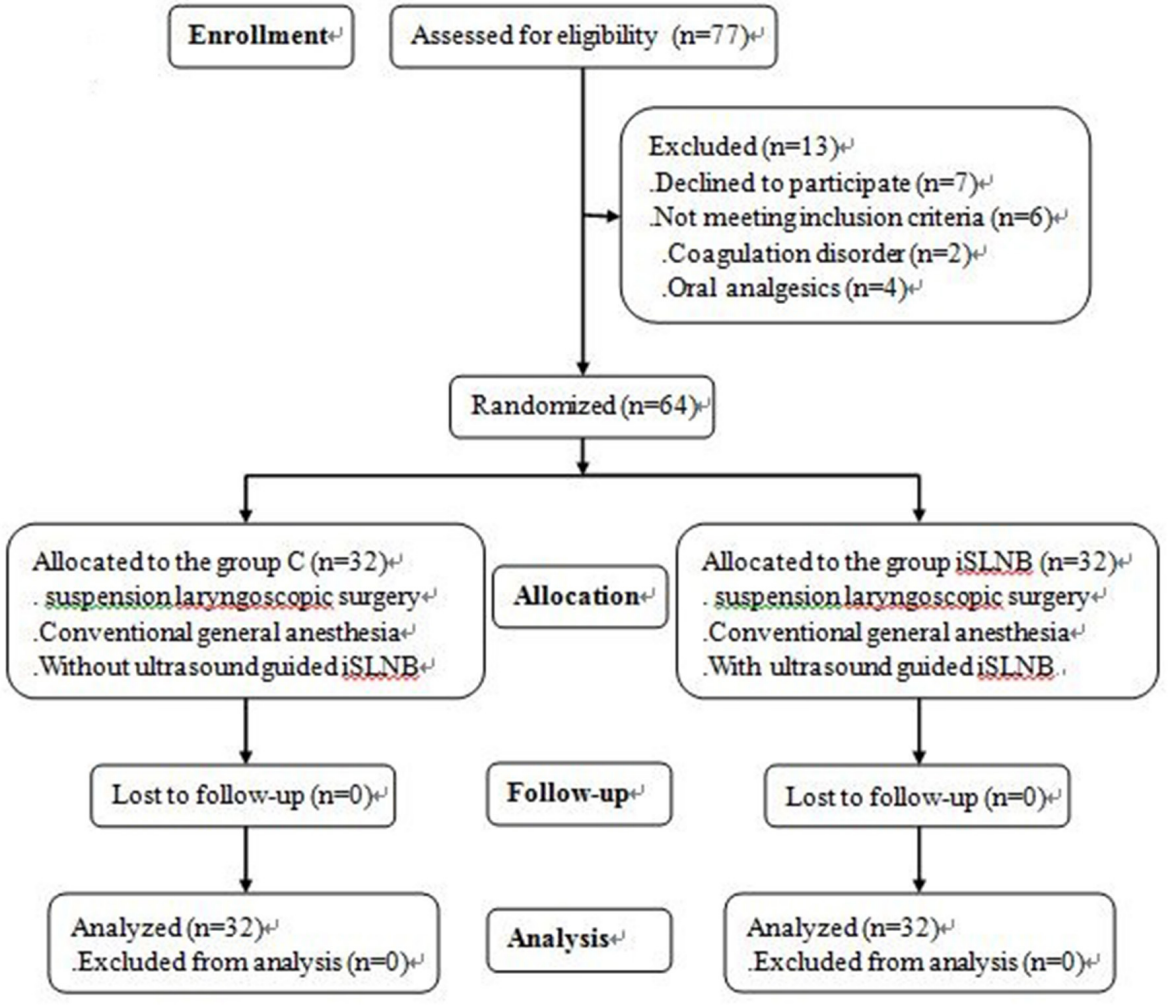
CONSORT flow diagram of this study iSLNB: internal branch of superior laryngeal nerve block.

The patients were assigned into two groups using a random number generator: the control group (C group) and the ultrasound-guided iSLNB group (iSLNB group). Group allocation was concealed to blind both the patients and the investigators who collected and analyzed the patient data (Figure 1).

### Study Protocol

After presenting to the operating room, all the patients were monitored routinely. Electrocardiogram (ECG), heart rate (HR), non-invasive blood pressure (NIBP), and pulse oxygen saturation (SpO_2_%), were performed continuously and recorded automatically at 5 min intervals. A bispectral index (BIS) monitor assessing the level of consciousness was also applied before general anesthesia induction. Anesthesia induction began with intravenous administration of 1 mg midazolam. Induction and maintenance of anesthesia were obtained with effect-site target control infusion of remifentanil and propofol. The target plasma effect-site concentration (Ce) values of propofol and remifentanil were set at 3–4 μg/ml and 3 ng/ml during induction, respectively. After loss of consciousness, cisatracurium (0.2 mg/kg) and 80 mg methylprednisolone were administered intravenously. After confirming muscle relaxation, endotracheal intubation was performed with a video laryngoscope, with a 6.5 mm internal diameter endotracheal tube for men and a 6.0 mm tube for women. Mechanical ventilation was performed with a tidal volume of 6–8 ml and respiratory rate of 10–15 per min to maintain P_ET_CO_2_ 35–45 mmHg. During anesthesia maintenance, the propofol target Ce was adjusted to keep the BIS between 40 and 60. Meanwhile, the remifentanil target Ce was titrated if HR and NIBP changed more than 20% of basic value. All patients completed tracheal extubation in the operating room and were transferred to the postanesthesia care unit after tracheal extubation.

**Figure 2:**
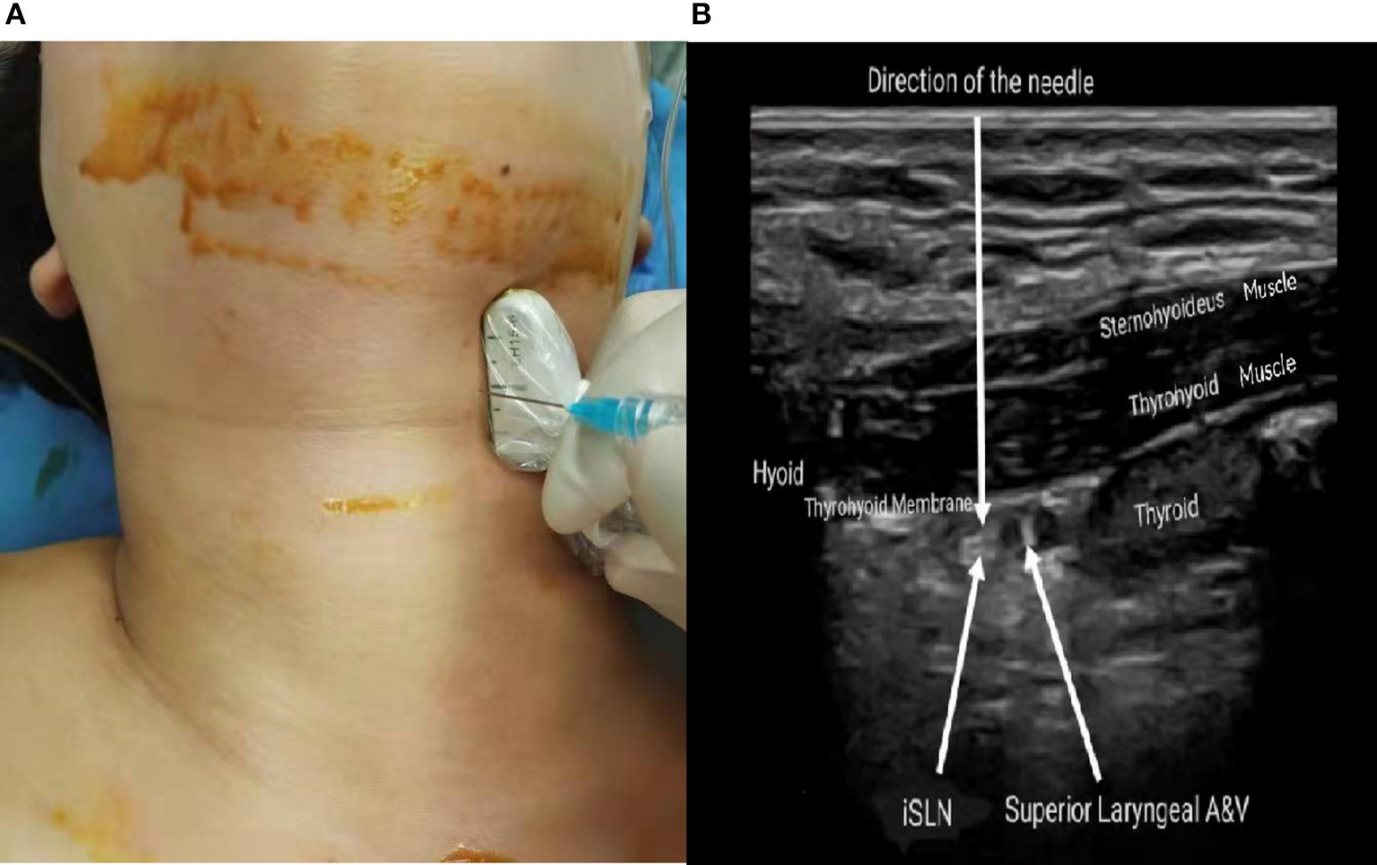
**(A)** Location of high frequency puncture probe and needle. **(B)** Ultrasound image of iSLNB.

After anesthesia induction, all patients were placed in the supine position with their neck adequately extended. Under complete aseptic precautions, a high-frequency “L” shape puncture probe (LH15-6, Wisonic Navi S, Shenzhen, China) was located over the submandibular area in a longitudinal orientation (Figure 2A). The thyroid cartilage and the greater horn of hyoid bone were hyperechoic signals on sonography. Thus, the thyrohyoid muscle and thyrohyoid membrane were identified between the two marks (Figure 2B). In sonography, the superior laryngeal artery was an important anatomic structure for the iSLN position. The block was performed bilaterally with the out-of-plane technique. The needle was inserted perpendicular to the probe until its tip was visible in the layer of interest and located to the target. Thus, 2 ml (0.2%) of ropivacaine was injected under the thyrohyoid membrane next to the superior laryngeal artery between two hyperechoic structures, whereas those in the C group received conventional general anesthesia only. Two surgeons, who were at an equal level of surgical competence, with at least 10 years of experience of laryngeal cancer resection with CO_2_ laser, performed this surgery.

### Outcomes

The primary outcome measures were the incidence and severity of POST and cough, which were evaluated at 0.5, 2, 6, and 24 h after tracheal extubation by an investigator who was blinded to the patients' assignment. The secondary outcome measures included HR and mean arterial pressure (MAP) at five time points: upon arrival in the operating room (T0), at endotracheal intubation (T1), at suspension laryngoscope insertion (T2), 5 min after suspension laryngoscope insertion (T3), at tracheal extubation (T4), and 5 min after tracheal extubation (T5). Blood glucose levels were measured at T0, T3, and T5. At the same time, the total operative duration, tracheal extubation duration, and incidence of postoperative complications were recorded, such as choking cough, aspiration, and dyspnea. The duration of tracheal extubation refers to the length of time from the end of the operation to the removal of the endotracheal tube.

The severity of POST and cough were assessed with a 4-grade scale (Table 1) (16); 0–I was considered mild, and II–III was considered severe. Because swallowing movement might increase POST, thus the severity of this complication was evaluated when patients were at rest without swallowing and when they were asked to swallow.

**Table 1:** Grading system for severity of postoperative score throat and cough.

**Grades**	**Severity of postoperative sore throat**	**Severity of postoperative cough**
0	No sore throat	No cough
I	Mild sore throat (complains of sore throat only upon inquiry)	Light or single cough
II	Moderate sore throat (complains of sore throat only)	More than one episode of unsustained (65 s) cough
III	Severe sore throat (severe pain associated with marked change in voice)	Sustained (65 s) and repetitive cough with head lift

*0-I was considered as mild, and II-III as severe*.

### Sample Size and Statistics Analysis

Based on our pilot study, the incidence of POST when undergoing suspension laryngoscopic surgery was ~80%, and we hypothesized that iSLNB would reduce the risk of POST by 20%. Thus, the sample size was calculated with a power of 0.8 and alpha error of 0.05. At least 25 subjects per group were needed. Assuming a 5% dropout rate and to increase the credibility of this study, 64 patients were recruited (32 subjects for each group).

Statistical analysis was performed using the IBM SPSS version 21 for Windows (IBM SPSS Inc., Beijing, China). Continuous variables are expressed as the mean ± SD, and categorical variables are presented as numbers (percentages). The Kolmogorov–Smirnov test was used to evaluate the normality of continuous variables, the Levene's test was used to evaluate the equality of variances, and their differences were analyzed with independent *t*-tests or Mann–Whitney *U*-tests where appropriate. Hemodynamic data from different time points were analyzed by one-way ANOVA. Categorical variables were evaluated via the Pearson χ^2^ test or Fisher exact test, where appropriate. *P* < 0.05 was considered as significant.

## Results

### General Data

A total of 77 patients were recruited, and 64 patients completed this study. There were no significant differences between the two groups relative to these demographic parameters (Table 2). Although the operative duration was similar in the two groups, the tracheal extubation duration of the iSLNB group was significantly shorter than that of the C group (*P* < 0.05).

**Table 2:** The demographic data, operative duration and tracheal extubation duration in two groups.

	**C (*n* = 32)**	**iSLNB (*n* = 32)**	***T***	***P***
Age (yrs)	54.8 ± 2.0	55.8 ± 1.9	0.385	0.701
Height (cm)	170.3 ± 7.2	170.2 ± 6.5	−0.036	0.971
Weight (kg)	69.4 ± 13.5	73.5 ± 14.4	1.185	0.240
BMI (kg/m^2^)	23.8 ± 3.4	25.3 ± 3.9	1.613	0.112
Operative duration (min)	53.6 ± 3.6	49.7 ± 3.0	−0.849	0.399
Extubation duration (min)^*^	12.8 ± 0.5	8.8 ± 1.7	−7.094	0.000

*C, control group; iSLNB, internal branch of superior laryngeal nerve block group; BMI, body mass index*.

* *The duration of tracheal extubation is the length of time from the emergence from anesthesia to the removal of the endotracheal tube*.

### The Incidences and Severity of POST and Cough

As shown in Table 3, iSLNB could significantly reduce the incidence and severity of POST and cough within 2 h after tracheal extubation, regardless of the presence or absence of swallowing. Additionally, iSLNB also ameliorated POST with swallowing 6 h after tracheal extubation. Twenty-four h after tracheal extubation, POST and cough caused by intubation and suspension laryngoscopy had completely resolved in the two groups.

**Table 3:** The incidences and severity of POST and cough.

	**0.5 h**		**2 h**		**6 h**		**24 h**	
	**C**	**iSLNB**	**C**	**iSLNB**	**C**	**iSLNB**	**C**	**iSLNB**
**Resting POST (*****n*** **=** **32 in each group)**								
Mild	2	32	11	32	32	32	32	32
Severe	30	0	21	0	0	0	0	0
χ^2^	56.471		31.256					
*P*	0.000		0.000					
**POST with swallow (*****n*** **=** **32 in each group)**								
Mild	0	31	0	32	24	32	32	32
Severe	32	1	32	0	8	0	0	0
χ^2^	60.121		64.000		9.143			
*P*	0.000		0.000		0.005			
**Postoperative cough (*****n*** **=** **32 in each group)**								
Mild	12	32	31	32	32	32	32	32
Severe	20	0	1	0	0	0	0	0
χ^2^	29.091		1.016					
*P*	0.000		1.000					

*C, control group; iSLNB, internal branch of superior laryngeal nerve block group; POST, postoperative sore throat*.

### Hemodynamics at Different Time Points in the Two Groups

Compared to the C group, except at T0, the iSLNB group had significantly reduced HR and MAP (Table 4).

**Table 4:** The hemodynamics and levels of blood glucose in two groups.

	***N* = 32**	**T0**	**T1**	**T2**	**T3**	**T4**	**T5**
HR (bpm)	C	72.2 ± 11.3	87.6 ± 10.8^*^	80.2 ± 13.1^*^ab	80.8 ± 11.8^*^ab	89.4 ± 9.7^*^	83.0 ± 11.1^*^b
	iSLNB	69.3 ± 8.9	72.4 ± 8.2	66.6 ± 8.3ab	69.4 ± 8.5	70.9 ± 6.1	69.3 ± 8.5
	*T*	−1.156	−6.350	0.111	−4.452	−9.083	−5.525
	*P*	0.252	0.000	0.000	0.000	0.000	0.000
MAP (mmHg)	C	97.4 ± 12.6	103.1 ± 12.6	100.0 ± 14.2	98.9 ± 14.0	100.5 ± 16.2	98.6 ± 14.1
	iSLNB	98.5 ± 9.7	91.0 ± 12.3^*^	88.6 ± 13.8^*^	85.5 ± 10.9^*^	88.8 ± 11.1^*^	87.5 ± 12.3^*^
	*t*	0.389	−3.872	−3.257	−4.288	−3.357	−3.342
	*P*	0.699	0.000	0.002	0.000	0.001	0.001
Blood glucose (mmol/L)	C	5.5 ± 1.0			5.9 ± 1.0		6.1 ± 1.2
	iSLNB	5.4 ± 0.9			5.4 ± 0.8		5.4 ± 0.8
	*t*	−0.604			−2.327		−2.806
	*P*	0.548			0.023		0.007

*C, control group; iSLNB, internal branch of superior laryngeal nerve block; HR, heart rate; MAP, mean arterial pressure*.

* *Compared to T0, P < 0.05. a compared to T1, P < 0.05. b compared to T4, P < 0.05. T0: arriving operating room, T1: endotracheal intubation, T2: suspension laryngoscope insertion, T3: 5 min after suspension laryngoscope insertion, T4: tracheal extubation, T5: 5 min after tracheal extubation*.

In the C group, compared to basic HR (T0), the HRs at other time points were significantly increased (*P* < 0.05). Although the MAPs of the C group at different time points were not significantly different, the MAPs of the other time points were slightly increased compared with those at T0 (*P* > 0.05). The HR at T1 was significantly higher than those at T2 and T3 (*P* < 0.05), and the HR at T4 was significantly higher than those at T2, T3, and T5 (*P* < 0.05).

In the iSLNB group, the HRs at T1 and T4 were significantly higher than that at T2 (*P* < 0.05). The MAPs at other time points were significantly lower than that at T0 (*P* < 0.05).

### Levels of Blood Glucose at Different Time Points in the Two Groups

Internal branch of superior laryngeal nerve block also significantly reduced the levels of blood glucose at T3 and T5 compared with the control (*P* < 0.05) (Table 4).

In the present study, there were no other complications, such as dyspnea or choking cough.

## Discussion

This study demonstrated that ultrasound-guided iSLNB with low concentration of ropivacaine could not only ameliorate the incidence and severity of POST and cough, but also stabilize the hemodynamics of patients undergoing endotracheal intubation, tracheal extubation, and insertion and maintenance of a rigid suspension laryngoscope.

Postoperative sore throat is the most common complaint in patients undergoing laryngoscopic surgery (17), and it is a prevalent complication following endotracheal intubation (18). Additionally, it is associated with tracheal extubation, which is another important factor contributing to the incidence of POST (19). Therefore, POST is not only a postoperative complication but also an intubation- and extubation-related complication. These procedures might produce subglottic injury and upper airway mucosal injury that induce POST (20). Although POST appears short-lived and usually resolves within a week or less, it should be viewed as a preventable side effect in the era of early enhanced recovery surgery in which patients return to full function as early as possible (21). In this study, most patients in both groups recovered from tracheal extubation within 6 h and recovered completely within 24 h.

Superior laryngeal nerve block is known to have great value in laryngopharyngeal surgery (22). Recently, it was also proven to improve POST after general anesthesia (23). In patients undergoing bronchoscopy examination, this technique could increase comfort and reduce complications, such as hypoxemia and cough (15). In the present study, iSLNB obviously ameliorated the incidence and severity of POST and cough, regardless of whether patients were swallowing, especially within 2 h after tracheal extubation. This supported the value of SLNB mentioned above. However, this result was also consistent with a study of SLNB in endoscopic laryngeal surgery (16), in which patients with SLNB had a much lower incidence of POST and cough.

The endotracheal intubation procedure can lead to sympathetic nervous system activation that causes an unstable hemodynamic response (24). In most patients, these hemodynamic responses are transient; however, in patients with cardiovascular and/or cerebrovascular diseases, these responses might produce dangerous complications (25). Patients undergoing suspension laryngoscopic surgery encounter the same cardiac reaction secondary to the sympathetic reflex and release of catecholamines, even though the stimulation is much more intensive and is sustained much longer than the transient process of endotracheal intubation (26, 27). Paltura et al. demonstrated that aerosolized lidocaine could inhibit the sympathetic reflex and block the SLN to effectively improve hemodynamics (27), and the effects of SLNB were proven in our study again.

In this study, the levels of blood glucose were significantly reduced by iSLNB. Blood glucose, as a metabolic substrate, is closely associated with the acute stress response (28). We speculated that iSLNB reduced the levels of blood glucose by inhibiting the activation of the sympathetic nerve system and acute stress response secondary to endotracheal intubation, suspension laryngoscope manipulation, and tracheal extubation. This might explain why the tracheal extubation duration of the iSLNB group was significantly shorter than that of the control group. Perhaps sympathetic suppression via iSLNB was able to decrease the need for anesthetics, which could be beneficial to patient recovery but was not examined in this study. Because there are few studies on the relationship between iSLNB and the duration of anesthesia recovery, this relationship might be included in our further studies.

Our study demonstrated that HR and MAP after endotracheal intubation and tracheal extubation were slightly higher than those after suspension laryngoscope manipulation, although there was no significant difference among these data. These results were contrary to our primary hypothesis that stimulation caused by suspension laryngoscopy was much more intensive than that caused by endotracheal intubation or tracheal extubation. An explanation might be that endotracheal intubation was completed without iSLNB. However, it remained unclear why HR and MAP were higher during tracheal extubation than those after suspension laryngoscope manipulation; this result was unexpected.

### Limitations

In our study, there are some limitations that should be considered when reviewing the results. For example, although this is a prospective randomized controlled study, it is a single-center trial with a small sample size, which inevitably lacks external validity. Second, lidocaine was used in most studies regarding SLNB, and we should add another group of lidocaine block to compare their different effects. Third, this study lacks long-term follow-up. All of these factors should be considered in future studies, which should have increased sample sizes and observations of long-term prognosis, for example, recrudescence, and mortality of tumors. Additionally, different volumes and concentrations of ropivacaine should be explored further to determine the optimal dose and concentration. Unsuitable nerve block might lead to severe complications (29); therefore, further study is important to avoid these complications. Finally, ~16.9% of patients were lost in the present study; this should be resolved for future study.

## Conclusion

Postoperative sore throat and cough were the most frequent complications after suspension laryngoscopic surgery with endotracheal intubation, and these manipulations might cause cardiovascular responses secondary to sympathetic nerve system activation. The ultrasound-guided iSLNB could obviously ameliorate these complications and make hemodynamic changes more stable without major complications.

## Data Availability Statement

The raw data supporting the conclusions of this article will be made available by the authors, without undue reservation.

## Ethics Statement

The studies involving human participants were reviewed and approved by Ethics Committee of Beijing Tongren Hospital, Capital Medical University. The patients/participants provided their written informed consent to participate in this study.

## Author Contributions

YB and JX designed this clinical trial and wrote the primary article. HW implemented all anesthesia protocols for the enrolled patients. YZ and QZ were responsible for these patients' treatment including operation. GW advised this trial and assisted with the writing in English. All authors contributed to the article and approved the submitted version.

## Funding

This work was supported by the Beijing Hospitals Authority Clinical Medicine Development of Special Funding Support, ZYLX202103.

## Conflict of Interest

The authors declare that the research was conducted in the absence of any commercial or financial relationships that could be construed as a potential conflict of interest.

## Publisher's Note

All claims expressed in this article are solely those of the authors and do not necessarily represent those of their affiliated organizations, or those of the publisher, the editors and the reviewers. Any product that may be evaluated in this article, or claim that may be made by its manufacturer, is not guaranteed or endorsed by the publisher.

## Abbreviations

BIS: Bispectral index
Ce: Concentration effect-site
HR: Heart rate
iSLNB: Internal branch of superior laryngeal nerve block
MAP: Mean arterial pressure
POST: Postoperative sore throat
SLN: Superior laryngeal nerve
SLNB: Superior laryngeal nerve block.
